# An Ontology-Based Approach for Consolidating Patient Data Standardized With European Norm/International Organization for Standardization 13606 (EN/ISO 13606) Into Joint Observational Medical Outcomes Partnership (OMOP) Repositories: Description of a Methodology

**DOI:** 10.2196/44547

**Published:** 2023-03-08

**Authors:** Santiago Frid, Xavier Pastor Duran, Guillem Bracons Cucó, Miguel Pedrera-Jiménez, Pablo Serrano-Balazote, Adolfo Muñoz Carrero, Raimundo Lozano-Rubí

**Affiliations:** 1 Medical Informatics Unit Hospital Clínic de Barcelona Barcelona Spain; 2 Clinical Foundations Department Universitat de Barcelona Barcelona Spain; 3 Fundació Clínic per a la Recerca Biomédica Barcelona Spain; 4 Data Science Unit Hospital 12 de Octubre Madrid Spain; 5 Direction of Planification Hospital 12 de Octubre Madrid Spain; 6 Unit of Investigation in Telemedicine and Digital Health Instituto de Salud Carlos III Madrid Spain

**Keywords:** health information interoperability, health research, health information standards, dual model, secondary use of health data, Observational Medical Outcomes Partnership Common Data Model, European Norm/International Organization for Standardization 13606, health records, ontologies, clinical data

## Abstract

**Background:**

To discover new knowledge from data, they must be correct and in a consistent format. OntoCR, a clinical repository developed at Hospital Clínic de Barcelona, uses ontologies to represent clinical knowledge and map locally defined variables to health information standards and common data models.

**Objective:**

The aim of the study is to design and implement a scalable methodology based on the dual-model paradigm and the use of ontologies to consolidate clinical data from different organizations in a standardized repository for research purposes without loss of meaning.

**Methods:**

First, the relevant clinical variables are defined, and the corresponding European Norm/International Organization for Standardization (EN/ISO) 13606 archetypes are created. Data sources are then identified, and an extract, transform, and load process is carried out. Once the final data set is obtained, the data are transformed to create EN/ISO 13606–normalized electronic health record (EHR) extracts. Afterward, ontologies that represent archetyped concepts and map them to EN/ISO 13606 and Observational Medical Outcomes Partnership Common Data Model (OMOP CDM) standards are created and uploaded to OntoCR. Data stored in the extracts are inserted into its corresponding place in the ontology, thus obtaining instantiated patient data in the ontology-based repository. Finally, data can be extracted via SPARQL queries as OMOP CDM–compliant tables.

**Results:**

Using this methodology, EN/ISO 13606–standardized archetypes that allow for the reuse of clinical information were created, and the knowledge representation of our clinical repository by modeling and mapping ontologies was extended. Furthermore, EN/ISO 13606–compliant EHR extracts of patients (6803), episodes (13,938), diagnosis (190,878), administered medication (222,225), cumulative drug dose (222,225), prescribed medication (351,247), movements between units (47,817), clinical observations (6,736,745), laboratory observations (3,392,873), limitation of life-sustaining treatment (1,298), and procedures (19,861) were created. Since the creation of the application that inserts data from extracts into the ontologies is not yet finished, the queries were tested and the methodology was validated by importing data from a random subset of patients into the ontologies using a locally developed Protégé plugin (“OntoLoad”). In total, 10 OMOP CDM–compliant tables (“Condition_occurrence,” 864 records; “Death,” 110; “Device_exposure,” 56; “Drug_exposure,” 5609; “Measurement,” 2091; “Observation,” 195; “Observation_period,” 897; “Person,” 922; “Visit_detail,” 772; and “Visit_occurrence,” 971) were successfully created and populated.

**Conclusions:**

This study proposes a methodology for standardizing clinical data, thus allowing its reuse without any changes in the meaning of the modeled concepts. Although this paper focuses on health research, our methodology suggests that the data be initially standardized per EN/ISO 13606 to obtain EHR extracts with a high level of granularity that can be used for any purpose. Ontologies constitute a valuable approach for knowledge representation and standardization of health information in a standard-agnostic manner. With the proposed methodology, institutions can go from local raw data to standardized, semantically interoperable EN/ISO 13606 and OMOP repositories.

## Introduction

The term primary use of health data encompasses the generation and use of data within the context of individual health care in hospitals and physicians’ offices to serve direct care needs [1]. The term secondary use of health data is defined by the American Medical Informatics Association as “non-direct care use of PHI [personal health information] including but not limited to analysis, research, quality/safety measurement, public health, payment, provider certification or accreditation, and marketing and other business including strictly commercial activities” [2]. Although they can be further categorized [3], one of the main types of secondary uses is research.

Clinical data sharing for research is highly relevant from a scientific, economic, and ethical perspective [4]. The overwhelming increment in the volume of available data is directly related with the emergence of a new paradigm of scientific methodology in which massive amounts of data are processed and analyzed for obtaining knowledge through machine learning and data mining algorithms [5].

Despite the growth of big data technologies and the use of artificial intelligence, in order to discover new knowledge from data, they must be correct and in a consistent format, which requires a great amount of resources for cleaning, binding, and organizing them. The semantics of data is a key component regarding the aforementioned challenges. To use the electronic health record (EHR) data for different projects, it must maintain its semantics and context, independently of any particular use case. This is especially important in research, where EHR reuse processes are often based on black boxes on which the final data customer is unaware of how the data uploaded to their research database were recorded, extracted, and transformed [6].

A common health information standard should be used in both primary and secondary use to share clinical information in a way that it can be unequivocally interpreted, both syntactically and semantically, by 2 or more systems. European Norm/International Organization for Standardization (EN/ISO) 13606 is a health information standard that seeks to define a rigorous and stable architecture for communicating the health records of a single patient, preserving the original clinical meaning. It is based on a dual model that includes a reference model (RM; with the necessary components and their constraints to represent EHR extracts) and an archetype model (AM; for the formalization of clinical-domain concepts according to the RM) [7,8]. Archetypes allow the formal representation of the structure of clinical information and its meaning (through terminology binding) so that it is automatically processable by information systems.

Furthermore, the EN/ISO 13940 norm [9] provides a conceptual framework centered in the clinical process. This norm, based on a clinical perspective, defines the system of concepts that are necessary for achieving continuity in the caregiving process, including both the content and the context of the health activities. This ample norm defines the concepts relative to health care actors, health problems, sanitary activities, health care processes, sanitary planification, time-related concepts, responsibilities, and information management.

Moreover, the Observational Medical Outcomes Partnership Common Data Model (OMOP CDM) defines a common format (data model), as well as a common representation (terminologies, vocabularies, and coding schemes), to allow systematic analyses of disparate observational databases using a library of standard analytic routines that have been written based on the common format [10]. The OMOP CDM is considered by several authors as the most adequate data model for sharing data in EHR-based longitudinal studies [11-13].

This paper describes the work carried out between Hospital Clínic de Barcelona (HCB), Hospital 12 de Octubre (H12O), and Instituto de Salud Carlos III (ISCIII), which seeks to consolidate clinical data of hospitalized patients with COVID-19 from different hospitals in joint repositories, structured with EN/ISO 13606 and then normalized according to the OMOP CDM.

The aim of this study is to design and implement a scalable methodology based on the dual-model paradigm and the use of ontologies to consolidate clinical data from different organizations in a standardized repository for research purposes without loss of meaning. This implies a series of particular objectives such as (1) to define a set of relevant clinical and biochemical variables of patients hospitalized with COVID-19, (2) to model a set of standardized archetypes based on EN/ISO 13606 to communicate such information, (3) to conceptually represent those clinical variables by means of ontologies, (4) to generate EN/ISO 13606–standardized EHR extracts of COVID-19 patients, and (5) to map and transform the source data to create OMOP CDM–compliant tables.

## Methods

### Ethical Considerations

This study was approved by the Hospital Clínic de Barcelona Ethics Committee for Investigation with Drugs (HCB/2018/0573).

### Cohort Inclusion Criteria

We included in this project patients with COVID-19 admitted to the emergency room (ER) or hospitalized between February 17, 2020 (beginning of the first wave in Spain), and February 15, 2022 (end of the sixth wave in Spain).

### Methodology

The following methodology comprises a series of steps in order to achieve the study’s objectives.

#### Step 1: Definition of Clinical Variables, Data Structures, and EN/ISO 13606 Archetypes

The first step consists in deciding the clinically relevant variables that should be included. Afterward, the data structures must be defined, including the fields, their descriptions, and the standardized terminologies and classifications to be used.

Since OMOP CDM is intended for secondary use of data (specifically, for biomedical research), its granularity is somewhat reduced when compared to raw data captured in hospitals. For this reason, the Medical Informatics Unit (MIU) at HCB decided to first standardize the data according to EN/ISO 13606, in order to have semantically interoperable EHR extracts with the maximum level of detail.

Therefore, the MIU at HCB and the Data Science Unit at H12O defined the EN/ISO 13606 archetypes to be used, modeled with the software LinkEHR [14] created by VeraTech for Health. The data types used are those established by the standard’s RM.

**Figure 1:**
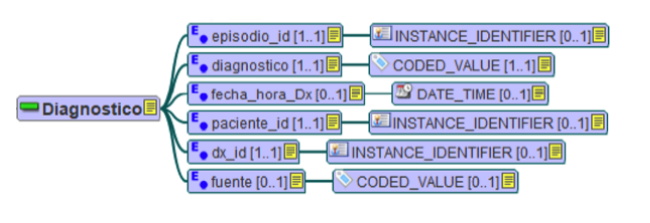
Mind map of the EN/ISO 13606 “diagnosis” archetype in Spanish, modeled with LinkEHR. The “diagnosis” entry has 6 elements: episode_id, diagnosis, diagnosis_datetime, patient_id, diagnosis_id, and source. Each of them has its corresponding data type. EN/ISO: European Norm/International Organization for Standardization.

This RM has multiple components, including the entry (a result of 1 clinical action, 1 observation, 1 clinical interpretation, or 1 intention) and its elements (the leaf node of the EHR hierarchy, containing a single data value). In our project, the archetypes modeled at the entry level of the RM were the following: diagnosis, episodes, limitation of life-sustaining treatment, administered medication, cumulative drug dose, prescribed medication, movements between units, clinical observations, laboratory observations, patients, health problems, and procedures. These archetypes were registered under a Creative Commons license (ID 2204210968527), so that any user who follows the license terms can share and adapt them [15]. Figure 1 shows a mind map of the diagnosis entry archetype as an example.

#### Step 2: Identification of Data Sources and Extract, Transform, and Load

Afterward, the corresponding data sources must be identified, in order to carry out the extract, transform, and load (ETL) process. In our case, these sources were (1) structured data from HCB’s health information system (HIS), SAP; (2) unstructured data from HCB’s HIS. A collaborative work with Barcelona Supercomputing Center (BSC) allows for the recognition of clinical entities through natural language processing and its extraction as normalized structured data; (3) outpatient setting structured data from Agència de Qualitat i Avaluació Sanitàries de Catalunya.

Since the last 2 sources come from separate projects whose description is besides the objective of this paper, we will focus on the first one. Archetypes created in the previous step were used as templates for identifying data in the aforementioned sources. Periodic meetings were held with the Information Technology Department at HCB to identify the location of the data and the transformations needed to obtain the structured data defined in the previous step. Once this was achieved, the tables were loaded into a MySQL database hosted on a dedicated server of the MIU.

#### Step 3: Creation of EN/ISO 13606 EHR Extracts From Source Data

Once the final data set is obtained, data must be transformed to create EHR extracts normalized according to EN/ISO 13606. This transformation includes mapping of local variables to standardized nomenclatures and classifications (Systematized Nomenclature of Medicine—Clinical Terms (SNOMED CT), International Classification of Diseases 10—Clinical Modification (ICD-10-CM), Logical Observation Identifiers Names and Codes (LOINC)), assigning readable descriptions to local codes, and categorizing certain concepts (eg, grouping hospital units according to the level of care).

This process is performed by mapping archetypes to the implicated information systems, without the need to modify them. This approach allows the automation of data extraction and the reuse of this methodology for other use cases with very little effort, which constitutes one of the great advantages of dual-model strategies.

**Figure 2:**
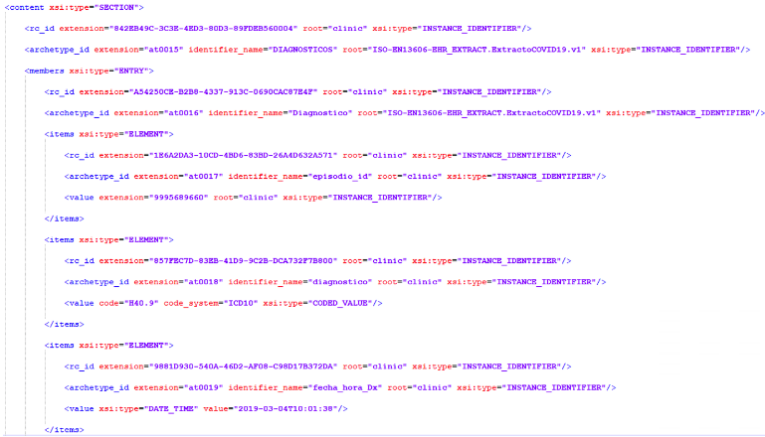
Anonymized, normalized EN/ISO 13606 EHR extract of diagnosis in Spanish. EHR: electronic health record; EN/ISO: European Norm/International Organization for Standardization.

In our case, we carried out this process with the help of VeraTech for Health, our technical partners, using LinkEHR, thus creating extracts on our dedicated server and constituting an EN/ISO 13606 standardized clinical repository. Figure 2 shows a test example of an EN/ISO 13606 EHR extract (without real-patient data). In this extract, the ICD-10-CM code H40.9 (unspecified glaucoma) is being communicated, alongside its date and time of record and the ID of the clinical episode it pertains to.

#### Step 4: Creation of Ontologies

Traditionally, clinical concepts and the relationships between them have been poorly developed in HISs. The MIU at HCB developed OntoCR, an ontology-based clinical repository, conforming to EN/ISO 13606 standard [16,17]. The use of ontologies allows for the definition of a conceptual architecture centered on the representation of the clinical process, while the use of EN/ISO 13606 allows syntactic and semantic interoperability between systems. EN/ISO 13940 was also used to define the generic concepts needed to achieve continuity of care, representing both the content and the context of the health care services.

One of the main advantages of ontologies is their flexibility to perform changes with minimum use of resources, adapting to an ever-changing environment. Likewise, ontologies allow the addition of conceptual layers, thus mapping locally defined concepts to health information standards, facilitating the communication of information without loss of meaning.

A relational database (OWL-DB) is used for storing ontologies and instantiated data, designed according to the Web Ontology Language (OWL) specification [18]. The ontologies in this project were created using Protégé, a free, open-source ontology editor created by Stanford University that fully supports OWL and Resource Description Framework (RDF) specifications from the World Wide Web Consortium [19]. A plug-in developed by our team, the OWL-DB plugin, connects Protégé with the OWL-DB module at the storage level.

These ontologies were conceptualized in 3 different layers. The first one describes the concepts modeled in the archetypes, with the classes and properties that describe the data structure defined in the first phase. Data types according to the EN/ISO 13606 RM were used.

In the next layer, we used a locally created ontology that reproduces the EN/ISO 13606 RM and AM. By creating an additional ontology that maps the archetypal concepts to the EN/ISO 13606 model, we structured our data according to the standard. In this layer, each entry-level archetype is represented in a separate ontology.

**Figure 3:**
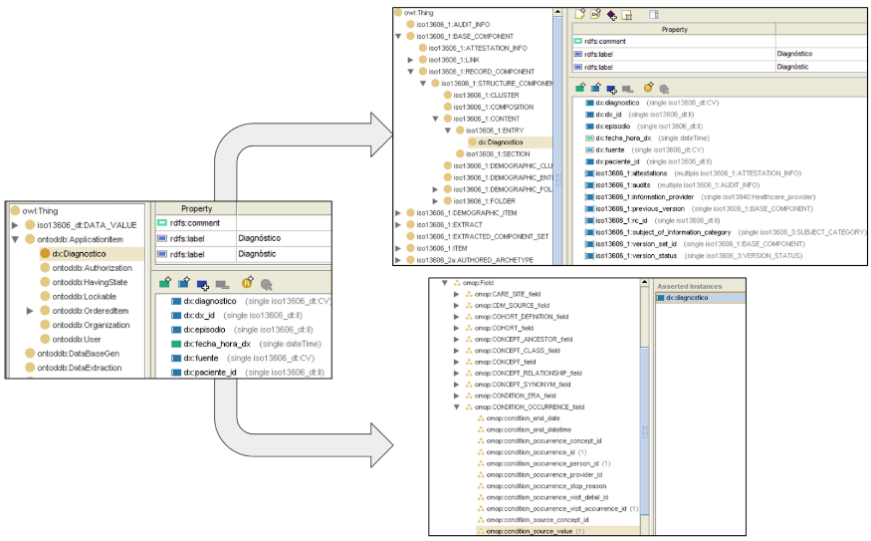
Ontologies of the archetype model of diagnosis (left) and its mapping to the EN/ISO 13606 structure (upper right) and the OMOP CDM (lower right) in Spanish, edited with Protégé software. EN/ISO: European Norm/International Organization for Standardization; OMOP CDM: Observational Medical Outcomes Partnership Common Data Model.

As with EN/ISO 13606, we created an ontology that models the OMOP CDM and afterward mapped archetypal concepts to the corresponding meta-class of the standard. So, the third layer consists of ontologies for each archetype that reproduce concepts according to the OMOP CDM structure. Figure 3 shows these 3 ontologies. The left image (ontology of the AM of diagnosis) depicts the class “Diagnosis” with its properties diagnosis, diagnosis_id, episode, diagnosis_datetime, source, and patient_id. In the upper-right image, a new ontology was created where the class “Diagnosis” was modeled as a subclass of “iso13606: Entry,” thus inheriting its properties defined in the RM. Finally, in the lower right image, a third ontology maps the property diagnosis with OMOP CDM’s metaclass “condition_source_value.”

Afterward, these ontologies must be loaded into a production environment of OntoCR so as to generate the structure that can receive instantiated data of patients and store it.

#### Step 5: Integration of EN/ISO 13606 Extracts Into the Ontology-Based Clinical Repository and Extraction of Data as OMOP CDM–Compliant Tables

Once the ontological structure is ready to receive the data, the EHR extracts must be inserted into the repository, thus incorporating the normalized, instantiated data. We initially explored the possibility of adapting a preexisting application programming interface (API) that was used for the same purpose in a previous project. However, the resources needed for its adaptation were significantly elevated, and its scalability reduced. Therefore, we decided to work on an application that identifies each archetype node within the extract and inserts it into its counterpart in the OWL file. This is facilitated by the representation in the ontologies of each archetype, their nodes, and the data types used (compliant with EN/ISO 13606).

Finally, data stored in the ontology-based clinical repository needs to be extracted through SPARQL queries, a language used for graph databases. Since archetypal concepts have been previously mapped to the OMOP CDM, by performing these queries, the extraction process is simplified. If there are cases in which data needs to be transformed to fit the CDM, such transformations can be included in the queries or carried out via SQL queries once relational tables are obtained.

**Figure 4:**
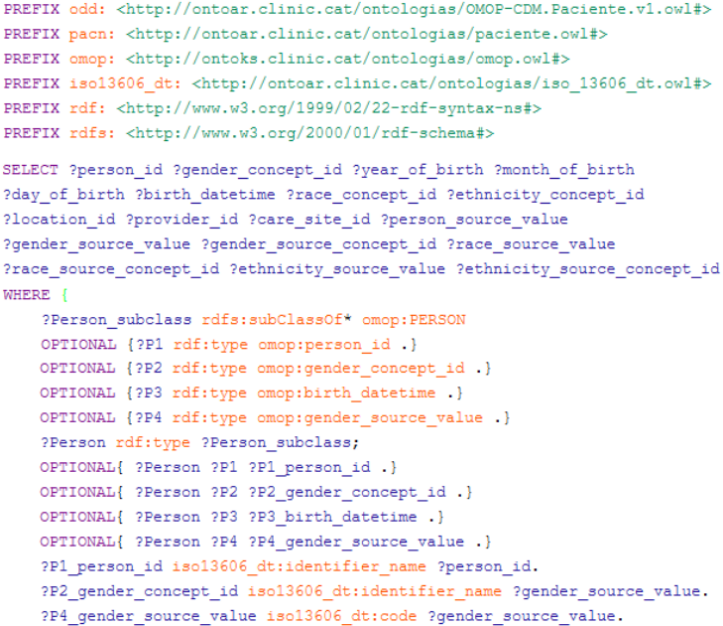
SPARQL query for the “Person” table of the OMOP CDM. OMOP CDM: Observational Medical Outcomes Partnership Common Data Model.

In Figure 4, a SPARQL query for extracting data for the OMOP CDM PERSON table is shown. Attributes that are not present in the ontological repository must still be included in the SELECT clause so as to create the corresponding table column without any instantiated data. Since EN/ISO 13606 data types were used in the extracts and modeled in the ontologies, they were also represented in the queries (see the lower lines of SPARQL code).

Data anonymization is performed by the IT department at this level using an institutional software solution. This way, EN/ISO 13606 extracts contain identified data that can be used for primary uses, while OMOP CDM tables are anonymized for secondary uses.

Once obtained, the anonymized data can be consolidated in a joint OMOP CDM repository with other institutions that use the same standard (in our case, H12O). OMOP CDM has a large number of tables, divided into 6 groups: standardized clinical data, standardized health system data, standardized derived elements, standardized health economics, standardized metadata, and standardized vocabularies. Our OMOP CDM repository contains the following tables, which are part of the standardized clinical data: “Condition_occurrence,” “Death,” “Device_exposure,” “Drug_exposure,” “Measurement,” “Observation,” “Observation_period,” “Person,” “Visit_detail,” and “Visit_occurrence.”

**Figure 5:**
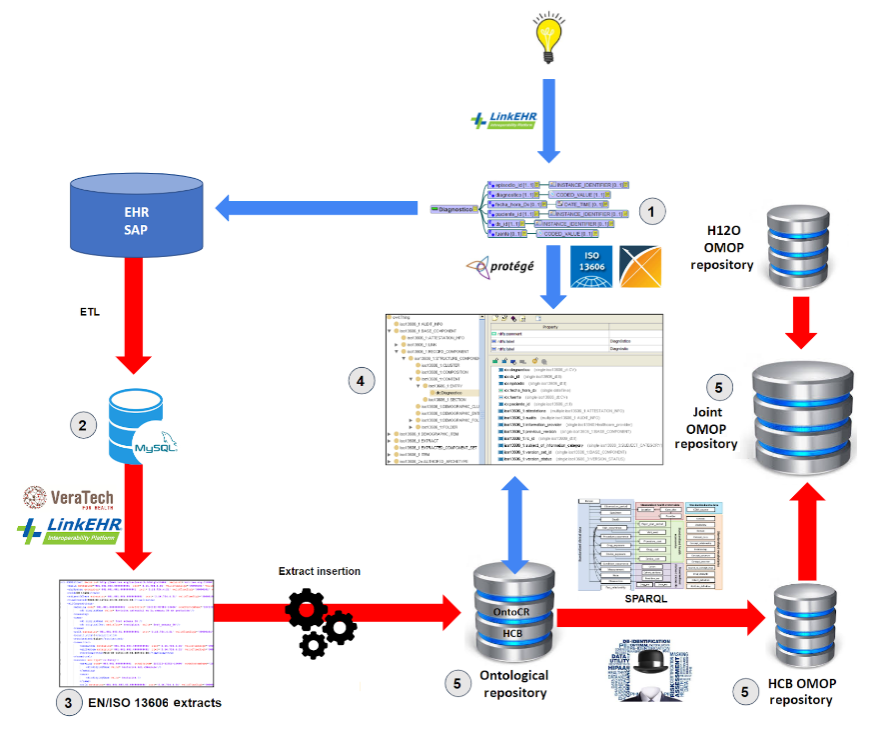
General overview of the process. Red arrows indicate data flow, while blue arrows indicate knowledge-related processes. Numbers indicate the deliverables within each step. EHR: electronic health record; EN/ISO: European Norm/International Organization for Standardization; ETL: extract, transform, and load; H12O: Hospital 12 de Octubre; HCB: Hospital Clínic de Barcelona; OMOP: Observational Medical Outcomes Partnership.

Figure 5 shows an overview of the whole process. The knowledge modeling starts with the creation of EN/ISO 13606 archetypes based on clinical concepts, which are then represented in ontologies that map them to EN/ISO 13606 RM and OMOP CDM. These ontologies are uploaded to OntoCR without instantiated patient data yet.

The data-related processes begin with the archetype-based extraction of raw data from our local system into a MySQL database and its transformation to create EN/ISO EHR extracts, which are then inserted into OntoCR via an application specifically developed for this project. SPARQL queries are performed against this ontological repository to obtain an OMOP CDM repository that is consolidated with H12O in a joint one.

## Results

### Methodology

The main deliverable of this project is the methodology described in the previous section. By following the aforementioned steps, any health care institution can go from local raw data to standardized, semantically interoperable EN/ISO 13606 and OMOP repositories. This methodology also led to the creation of 12 EN/ISO 13606-standardized archetypes that model important clinical variables in the ER and hospitalization settings, allowing the reuse of clinical information by using it in accordance with the Creative Commons terms.

### Ontologies

Another interesting result of this study is the development of the ontologies that represent OMOP CDM, as well as their mappings to EN/ISO 13606 AM and RM. This process was carried out by members of the MIU at HCB after carefully reading the pertinent documentation of these standards and designing the optimal way of using them to represent clinical concepts.

Furthermore, representing clinical variables by means of ontologies is another way of reusing clinical information. With the creation of new ontologies for each project at HCB, where we have developed the ontology-based clinical repository OntoCR, we continue to extend our clinical knowledge representation.

### EN/ISO 13606 Extracts

Table 1 shows the correspondence between EHR archetypes, OMOP CDM tables, number of extracts created throughout the study, and the number of COVID-19 patients they pertain to. We have included the diagnoses recorded in the episodes of the study period as well as the historical ones. Health problem entries are part of the aforementioned project with BSC to extract clinical entities from unstructured texts through natural language processing, so they will not be included in this table.

**Table 1:** Correspondence between EHR^a^ archetypes, OMOP CDM^b^ tables, number of extracts created throughout the study, and the number of patients they pertain to.

EHR archetype	OMOP CDM table	EN/ISO^c^ 13606 extracts, n	Patients, n
Patient	“Person”	6803	6803
Episode	“Visit_occurence”	13,938	6791
Diagnosis	“Condition_occurrence”	190,878	6799
Cumulative drug dose	“Drug_exposure”	262,770	5630
Administered medication	“Drug_exposure”	262,770	5630
Prescribed medication	“Drug_exposure”	341,986	5639
Movements between units	“Visit_detail”	47,817	6791
Clinical observation	“Measurement”	6,736,745	5973
Laboratory observation	“Measurement”	3,392,873	6001
Limitation of life-sustaining treatment	“Observation”	1298	1142
Procedure	“Procedure_occurrence”	19,861	4994

^a^EHR: electronic health record.

^b^OMOP CDM: Observational Medical Outcomes Partnership Common Data Model.

^c^EN/ISO: European Norm/International Organization for Standardization.

### OMOP CDM–Compliant Clinical Tables

We still do not have the final number of records in our OMOP tables, since the initial approach of adapting the preexisting API had to be replaced by the creation of the application that inserts data from the extracts into the ontologies. However, an OMOP database for a random small subset of patients was successfully created to test the queries and validate the methodology. This was performed using a locally developed Protégé plugin (“OntoLoad”) that imports a set of data from a relational database into the ontologies [17]. Table 2 describes the OMOP tables that were created.

**Table 2:** OMOP CDM^a^-compliant clinical tables created for a random small subset of patients.

OMOP CDM table	Patients, n	Records, n
“Condition occurrence”	121	864
“Death”	110	110
“Device_exposure”	3	56
“Drug_exposure”	106	5609
“Measurement”	3	2091
“Observation”	3	195
“Observation_period”	897	897
“Person”	922	922
“Visit_detail”	250	772
“Visit_occurrence”	897	971

^a^OMOP CDM: Observational Medical Outcomes Partnership Common Data Model.

## Discussion

### Principal Results

This study proposes a methodology for standardizing clinical data, thus allowing its reuse without any change in the meaning of the modeled concepts. Although the focus of this paper is health research, our methodology suggests that the data be initially standardized according to EN/ISO 13606 to obtain EHR extracts with a high level of granularity that can be used for any purpose, as previous studies have suggested [20]. Afterward, its transformation to OMOP CDM–compliant tables allows its consolidation in joint repositories for research purposes.

Although EN/ISO 13606 was chosen because of the operational mechanisms it offers for data exchange, due to the flexibility and standard-agnostic nature of our methodology, there is complete independence regarding any specific standard. Thus, by modeling ontologies of other standards and mapping them to local variables, we may, for example, carry out transformations between EN/ISO 13606, OpenEHR [21], Fast Healthcare Interoperability Resources (FHIR) [22], OMOP CDM, and Informatics for Integrating Biology and the Bedside (i2b2) [23] with the minimum use of resources and without the need for changes in the database structure. Health information standards such as EN/ISO 13606 and OpenEHR allow the modeling and formalization of clinical knowledge through their RMs and archetypes [24], and ontologies are precisely a tool for carrying out such tasks. This is what makes them ideal in the context of an implementation of a dual-model strategy, allowing the representation of concepts in the health domain, its standardization, and the storage of instantiated patient data.

Furthermore, ontologies provide several advantages for the conceptualization of entities in a domain. It explicitly represents domain knowledge, allows the application of inference processes, enables the reuse of domain knowledge, allows data aggregation, and detects new associations between concepts [17].

It is clear for us that loading normalized data onto clinical repositories (instead of ad hoc data loading) provides many benefits. It is possible to reuse the same interoperability standards used in health care, adapting them to this new paradigm [25]. This approach allows the availability of clinical data for further single- or multicenter research.

We would like to highlight the vital importance of continuous collaborative research. This study is framed within a continued line of research since 2009 between HCB, ISCIII, and H12O. In this line of collaborative research, a standardized and transparent process has been designed and implemented for obtaining standardized data models for research from EHR raw data. Hence, in the first stage, the basis for a semantically interoperable clinical information management system based on EN/ISO 13606 was defined, proving that clinical information residing in heterogeneous systems could be normalized, combined, and communicated without loss of meaning. In the second stage, a common information model that reflects the clinical process and the relationships between the clinical records components was developed. In the third stage, a normalized information model based on EN/ISO 13606 archetypes was implemented and applied to local information systems for specific clinical use cases. With this model, it is possible to construct and order information recovered from these complex systems for the exchange of integral health and social information of patients and to use it for secondary purposes.

### Comparison With Prior Work

Many of the requisites of clinical data repositories for primary use are common to those for secondary use, such as normalized clinical information models, controlled terminologies, identification of actors, and contextual information. Developments carried out for primary use repositories are also profitable for secondary uses, and the progresses derived from secondary uses accelerate the advances in shared clinical records. A lot of work has been reported in this field throughout the globe in the last years, which has led to developing policies, repository models and its application in the form of competitive projects [2,26,27].

It is very usual for researchers to resort to the generation of their own data for research and its manual introduction into data management systems. It is also quite common for them to use general purpose tools, particularly spreadsheets, as data management systems [28], while there is perception of a high need of additional support for the analysis of high volumes of data. This represents a significant problem, since these applications cannot guarantee the consistency of data, and they present difficulties for sharing and consolidating data and a limited capability of data exploitation.

Different methodologies have been proposed to create OMOP repositories from raw data. Some approaches are based on a simple mapping of local variables to their OMOP CDM counterparts, an alignment of vocabularies using the Athena tool provided by OHDSI and an ETL process through SQL scripts [29]. Other authors have proposed transforming source data to RDF, carrying out a semantic mapping (in some cases, using an ontological representation of OMOP CDM), and loading it to a data store [30,31].

Likewise, other standard-agnostic approaches have been reported in the literature. The ongoing INFOBANCO project of the Madrid Region [32] seeks to create a platform for the management, persistence, exchange, and reuse of health data focused on applying each health information standard for the purpose it was intended to, offering multiple interoperability and exploitation services suited for specific use cases [24]. Furthermore, the 3-pillar strategy of the Swiss Personalized Health Network [33] pursues a semantically interoperable clinical data landscape based on a multidimensional encoding of concepts, an RDF-based storage and transport of the instances of these concepts and a conversion of RDF to any target data model.

### Strengths and Limitations

This study has many strengths that are worth mentioning. On the one hand, it describes a real-world collaborative effort between 3 health care institutions in Spain to model, share, and consolidate standardized patient data. Furthermore, the standard-agnostic nature of the proposed methodology leads to a significant scalability, allowing transformation between different health information standards and common data models. The software used in our methodology (LinkEHR, Protégé, and Liferay) either have a free version or are open source, which make them accessible to low-income areas and institutions with limited funding for interoperability projects.

We must also mention the limitations of this study. First of all, the ontology-based clinical repository used in our institution was developed throughout many years, and it might not be a suitable approach for institutions that seek a rapid implementation of a methodology. This can limit the external validity of the study. Moreover, since the tool to insert data from standardized extracts into the ontologies is not ready yet, we still have not completed the creation of OMOP CDM tables. However, an OMOP CDM database for a small subset of patients was successfully created to test the queries and validate the methodology.

### Next Steps

The MIU team at HCB is working on creating the ontological representation of different health information standards (FHIR and OpenEHR) and CDMs (i2b2, International Cancer Genome Consortium Accelerating Research in Genomic Oncology (ICGC Argo) [34], and Clinical Data Interchange Standards Consortium (CDISC) [35]). This will extend the current metamodel and allow us to carry out multistandard transformations, which will also help us compare the performance of such standards for different scenarios.

### Conclusions

Semantic interoperability plays a very important role within HISs, providing meaning and clinical context to the clinical information and allowing for better clinical decision-making and research. This study has demonstrated that ontologies constitute a valuable approach for knowledge representation and standardization of health information in a standard-agnostic manner. With the proposed methodology, institutions can go from local raw data to standardized, semantically interoperable EN/ISO 13606 and OMOP repositories.

## Abbreviations

AM: archetype model
API: application programming interface
BSC: Barcelona Supercomputing Center
CDISC: Clinical Data Interchange Standards Consortium
EHR: electronic health record
EN/ISO 13606: European Norm/International Organization for Standardization
ER: emergency room
ETL: extract, transform, and load
FHIR: Fast Healthcare Interoperability Resources
H12O: Hospital 12 de Octubre
HCB: Hospital Clínic de Barcelona
HIS: health information system
i2b2: Informatics for Integrating Biology and the Bedside
ICD-10-CM: International Classification of Diseases 10—Clinical Modification
ICGC Argo: International Cancer Genome Consortium Accelerating Research in Genomic Oncology
ISCIII: Instituto de Salud Carlos III
LOINC: Logical Observation Identifiers Names and Codes
MIU: Medical Informatics Unit
OMOP CDM: Observational Medical Outcomes Partnership Common Data Model
OWL: Web Ontology Language
OWL-DB: Web Ontology Language database
RDF: Resource Description Framework
RM: reference model
SNOMED CT: Systematized Nomenclature of Medicine—Clinical Terms
